# Case report: Recombinant human epidermal growth factor gel plus kangfuxin solution in the treatment of aplasia cutis congenita in a case with Adams–Oliver syndrome

**DOI:** 10.3389/fsurg.2022.1072021

**Published:** 2023-01-11

**Authors:** Xiu-Fang Yang, Shang-Wen Shi, Kang Chen

**Affiliations:** ^1^ Department of Neonatology, Zhongshan Hospital Affiliated to Sun Yat-Sen University, Zhongshan, China; ^2^ Molecular Inspection Center, Zhongshan Hospital Affiliated to Sun Yat-Sen University, Zhongshan, China

**Keywords:** neonates, aplasia cutis congenita, *DLL4* gene, Adams–Oliver syndrome, case report

## Abstract

**Background:**

Aplasia cutis congenita is a congenital disorder with the absence of skin, muscle and(or) bone. It usually affects the scalp. The presence of a large scalp defect can be potentially serious when complicated with hemorrhage and infection. Early healing of this condition is beneficial to improve the prognosis of infants.

**Study case:**

A full-term newborn male was born with a round-shaped defect at the vertex of the scalp and skull (dimensions, 8 cm × 9 cm). The infant had a large deletion encompassing the 15.1 region of chromosome 15, including the *DLL4* gene. Genetic testing was positive for Adams–Oliver syndrome (AOS). After two months of recombinant human epidermal growth factor gel combined with kangfuxin solution therapy, the skin defects of the scalp healed remarkably. The infant had regular follow-up appointments. At the age of 5 months, the defect became smaller, hairless, and showed good granulation tissue. At 2 years of age, the child's Gesell Developmental Schedules was 70.

**Conclusion:**

Recombinant human epidermal growth factor gel combined with kangfuxin solution was a successful conservative treatment for an infant with a large scalp defect accompanied by AOS.

## Introduction

Aplasia cutis congenita (ACC) is a defect that can affect the skin, dermis, and even subcutaneous tissue (including muscle and bone) in neonates (1, 2). A scalp defect is the most common side effect in infants with ACC, particularly in those with Adams–Oliver syndrome [AOS (MIM 100300)]. The clinical manifestations of AOS include congenital hypoplasia of parietal skin, tricuspid regurgitation, ventricular septal defect, skull defect, short finger syndrome, short and toe deformity, some patients with marble-like skin and nail dysplasia (3–6). The cause of Adams–Oliver syndrome is unknown, maybe sporadic or hereditary. A scalp defect, combined with bleeding and infection, can lead to adverse outcomes in children. Proper management strategies for scalp defects are thus extremely important for improving prognosis. Due to the lack of consensus or evidence-based guidelines for ACC management, treatment strategies are often individualized.

We report on a family in which the son has Adams–Oliver syndrome. The son carries with a 625.9 kb deletion including *DLL4* (Delta Like Canonical Notch Ligand 4) gene (MIM 605185) which may cause AOS. The son with AOS has scalp defect and skull absence. The scalp defect of this case with AOS had been successfully treated by combining recombinant human epidermal growth factor gel with kangfuxin solution.

## Patient, clinical signs, and outcomes

**Figure 1:**
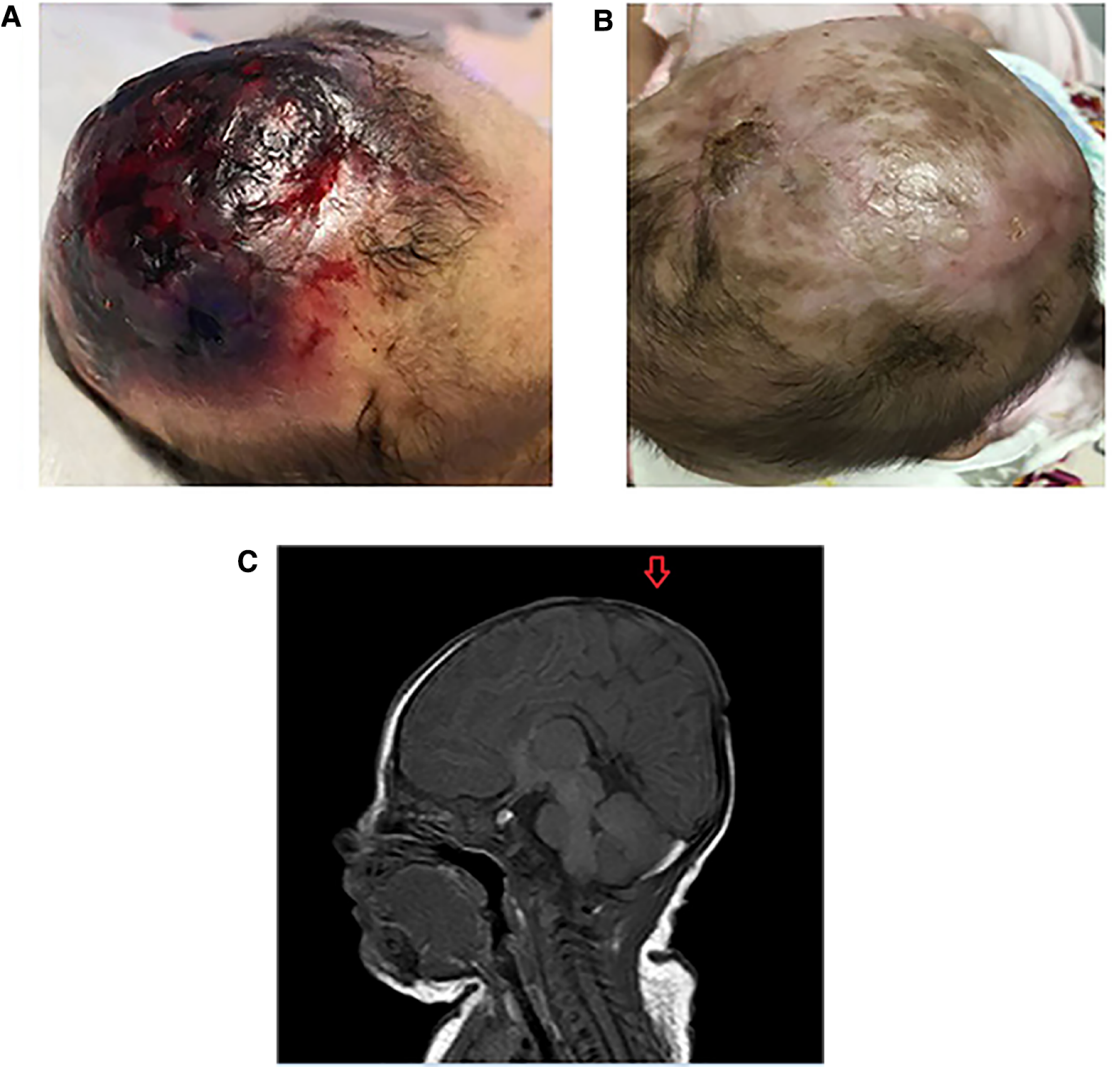
(**A**) The scalp defect of the patient with AOC at birth. (**B**) The scalp of the patient with AOC at 5 months old. (**C**) Cranial magnetic resonance imaging scan of the patient with aplasia cutis congenital (AOC). The scan shows a complete loss of continuity of the dermal, subcutaneous, and skull tissues (arrow).

A full-term male newborn with a round-shaped defect at the vertex of the scalp and skull (dimensions, 8 cm × 9 cm) was was born in the Obstetrics Department of Zhongshan Hospital Affiliated with Sun Yat-Sen University (also known as Zhongshan City People's Hospital) on May 10, 2019 (Figure 1A). The infant weighed 2.5 kg (−2SD) (38 weeks gestational age). The height of the infant was 48.6 cm (−1SD). Th head circumference was 33.2 cm (–1SD). The infant's mother experienced premature rupture of amniotic membrane for 11 h prior to giving birth and had gestational diabetes mellitus. Her examination showed no placental abnormalities, and no herpes simplex, varicella-zoster, rubella, or cytomegalovirus infections. The mother denied having been exposed to teratogenic drugs, radiation, or chemicals during pregnancy. The pregnancy of his mother was G1P1. There was no history of hypertensive disorder complicating pregnancy. There were no family histories of aplasia cutis congenita and other congenital malformations.The Apgar score of the infant was 10 at 1, 5, and 10 min after birth. He had no congenital malformation of the digestive tract, nor skeletal or congenital heart malformations. Peripheral blood, white blood cell, and blood gas analyses, as well as lactic acid, serum C-reactive protein, blood ammonia, and hepatobiliary biochemistry test results, were all normal. The blood cultures revealed no bacterial growth.

**Figure 2:**
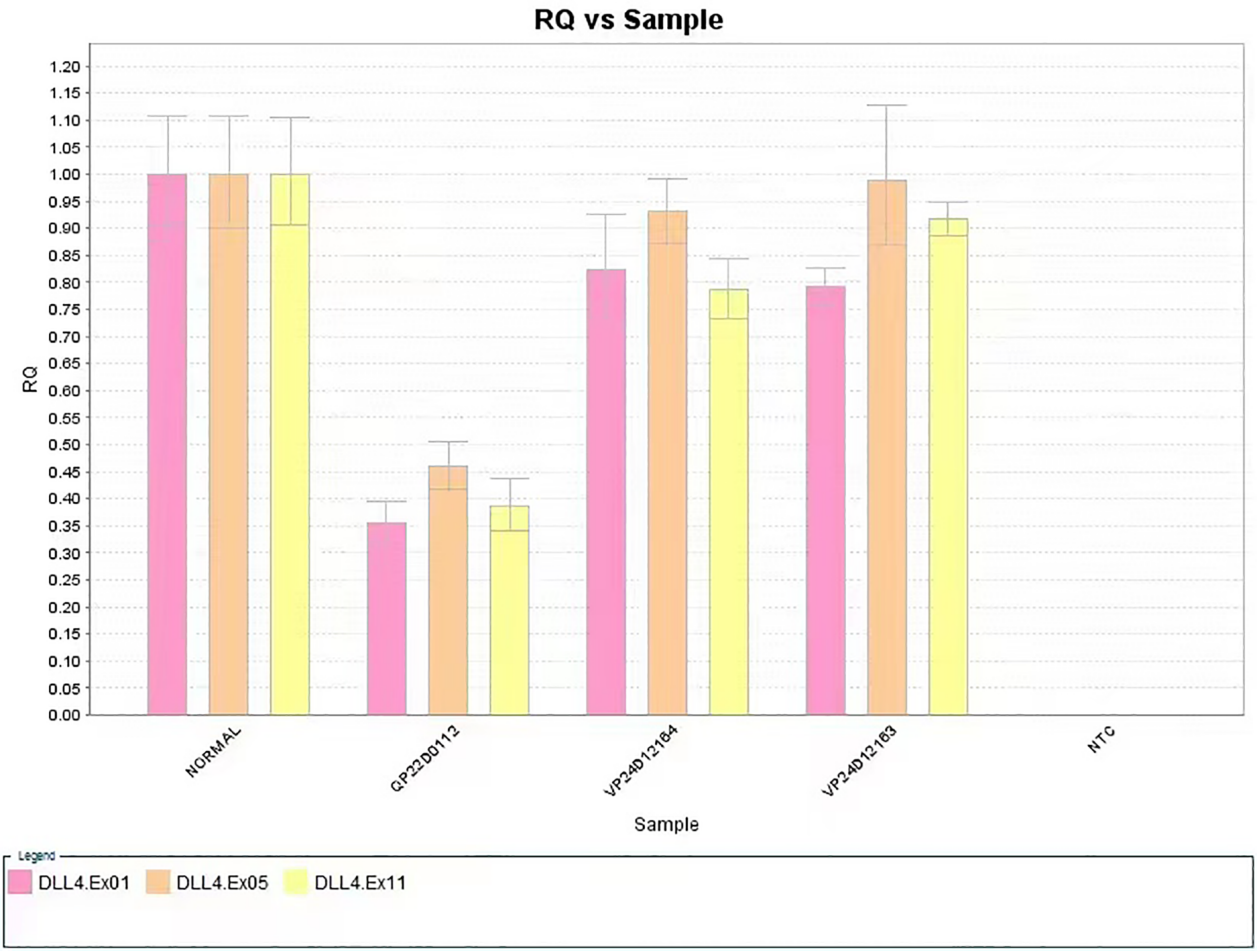
The large deletion of exon1, exon5, and exon11 of the *DLL4* gene of the case study infant (sample no. QP22D0112, the patient; Sample no. VP24D12164, the patient's father; Sample no. VP24D12163, the patient's mother).

The defect included scalp and skin layers and was confirmed by magnetic resonance imaging (MRI) examinations on May 13, 2019. The cranial MRI scan revealed complete loss of continuity of the dermal and subcutaneous tissues and skull (Figure 1B). Echocardiography, a chest x-ray, and abdominal ultrasonography revealed no apparent abnormalities. Peripheral blood chromosome karyotyping was 46, XY. The metaphases of the 20 cells were analyzed under the microscope. The list of organisms by chromosome count was normal. Chromosome structure was not broken, deletion, repeat, ectopic, inversion, ring and isobar chromosome aberrations. Genetic testing indicated a 625.9 kb deletion (Copy number variation 1) in the 15.1 (41043614-41669512) region of chromosome 15 of the infant, which involved in *DLL4, KIF21B, DNAJC17, ZFYVE19, SPINT1, VPS18* gene, etc. The deletion of *DLL4* gene was found to be related to the clinical manifestation of this infant through the GeneCards(the human gene database). The results of his parents showed that the copy number in this region was normal, which suggested that the heterozygous deletion of *DLL4* gene was new in the infant. The defect of *DLL4* gene is sporadic.The result of genetic qualitative dynamic mutation, verified by semi-quantitative polymerase chain reaction with a specific gene deletion, showed a large deletion of exon1, exon5, and exon11 of the *DLL4* gene of the infant, which can cause AOS (Figure 2). The *DLL4* gene is a protein coding gene. The deletion of *DLL4* gene may cause Adams–Oliver syndrome type 6 (MIM 616589). The child was diagnosed with AOS, based on his clinical presentation and the genetic examination.

**Figure 3:**
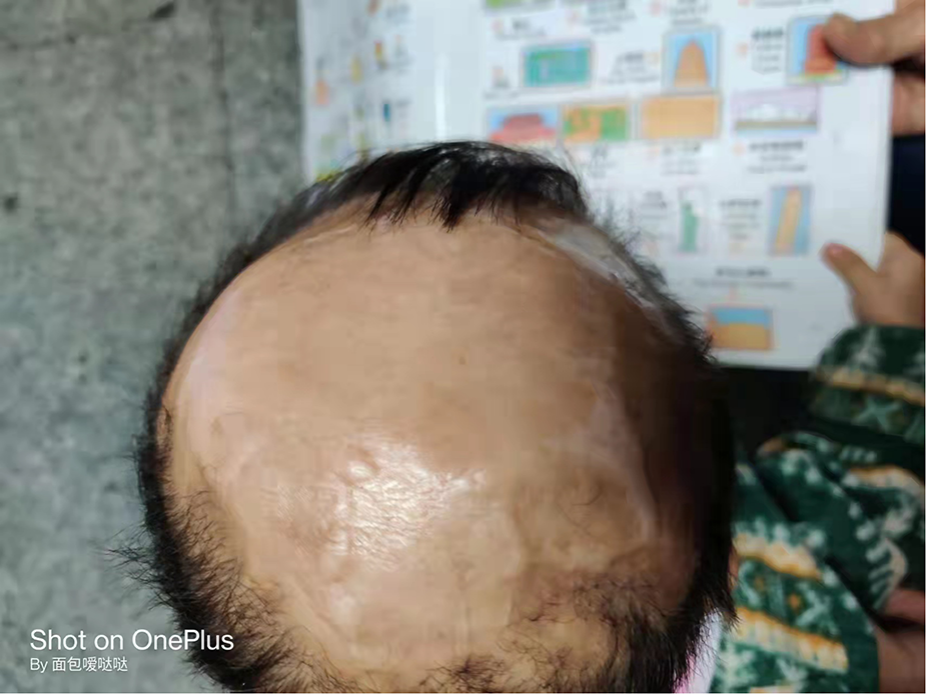
The scalp of the patient with aplasia cutis congenita at 2 years old.

The lesion of scalp was treated with normal saline cleansing, recombinant human epidermal growth factor gel, and kangfuxin solution for two months. Following daily local treatment, the wound gradually healed, and the lesion of scalp exhibited good epithelialization and eschar formation. The child attended regular follow-up visits. Complete epithelialization was achieved and the defect of scalp became hairless with good granulation tissue at five months (Figure 1C). At 2 years old, the scalp defect became smaller and hairless (Figure 3), and Gesell Developmental Schedules was 70. For large defects involving the skull, surgical intervention maybe the treatment of most ACC patients. However, his parents refused to reexamine the cranial MRI and surgery.

## Discussion

Adams–Oliver syndrome is a condition of scalp defects and/or limb deformities that were first reported by Addams and Oliver in 1945 (7). Its incidence is low and sporadic, but there is also a genetic tendency for developing the condition. To date, the etiology of AOS remains unknown. Vascular injury during embryogenesis and disruption of the blood supply to developmental structures, caused by placental thrombosis, has been identified as possible mechanisms of AOS (8, 9).

Gene mutations (*ARHGAP31*, *DLL4*, *NOTCH1*, *RBPJ*, *DOCK6*, and *EOGT*) are associated with AOS (10–12). The molecular mechanism of this syndrome, based on gene mutation, is guanosine-5′-triphosphate (GTP) being a primer for deoxyribonucleic acid replication, a 5′-guanosine monophosphate provider for transcription, and an energy carrier as part of the tricarboxylic acid cycle process (13). Guanosine-triphosphate-binding protein is a large family of hydrolases that can bind and hydrolyze GTP in a highly conserved G-domain (14). These proteins are important for signal transduction, protein biosynthesis, and protein and vesicle transport, respectively.

The results of proband gene detection indicated a large deletion in the 15.1 region of chromosome 15, including the *DLL4* gene. The protein encoded by the *DLL4* gene is part of the Notch signal transduction pathway (15). The *DLL4* protein is essential for vascular development; patients with a *DLL4* gene deletion may develop ACC and extremity defects, which may result from the abnormal development of small blood vessels, thrombosis, and vascular obstruction (16).

A diagnosis of AOS is based on the clinical presentation of the scalp and cranial bone defects, as well as limb anomalies that are visible following the birth of an infant. Other presentations include a cleft lip or palate, growth retardation, cardiovascular abnormalities, urinary system abnormalities, and intellectually disabled (4). The current case included scalp and skull defects, as well as intellectually disabled. The scalp defect was large, and if not treated correctly, may have affected the healing of the skin defect, prolonged the course of treatment, and increased the incidence of infection and septic shock (17).

Some infants with severe ACC require skin grafting to prevent bleeding and infection (18, 19). Kangfuxin solution (*Periplaneta americana L*. extract) is used for trauma wounds, ulcers, fistulas, burns, scalds, and bedsores (20, 21). The current authors combined recombinant human epidermal growth factor gel with kangfuxin solution in an external application to successfully treat a scalp defect wound.

In general, cutis aplasia with AOS is a potentially life-threatening disease. Surgery, combined with conservative treatment, is an important approach for treating large skin defects in this context. Based on the current case, we found that recombinant human epidermal growth factor gel combined with kangfuxin solution worked as a successful conservative treatment for a large scalp defect of an infant with AOS and did not cause any serious complications.

## Data Availability

The original contributions presented in the study are included in the article/Supplementary Material, further inquiries can be directed to the corresponding author.
